# O-37 - PUBLIC HEALTH INTELLIGENCE: PROTECTING THE UK PUBLIC FROM EMERGING INFECTIOUS THREATS

**DOI:** 10.1093/pubmed/fdag046.033

**Published:** 2026-07-09

**Authors:** Dr Michael Reynolds, Dr Sherine Thomas

**Affiliations:** UK Health Security Agency; UK Health Security Agency

## Abstract

**Background:**

Public health intelligence underpins the UK’s ability to anticipate, detect, and respond to infectious disease threats. It encompasses the systematic identification, assessment, and communication of signals that may indicate emerging or escalating health risks to public health. Signal verification is strengthened through international collaboration, providing access to global data, specialist expertise, and contextual insight.

**Objectives:**

To describe the role of integrated public health intelligence processes in supporting rapid detection, verification, and assessment of infectious threats, and to demonstrate how these processes contribute to effective preparedness and response.

**Methods:**

Signals detected through routine event-based surveillance and intelligence-gathering activities are assessed for relevance, credibility and potential impact. Verification is strengthened through collaboration with international partners, enabling timely data sharing and contextual interpretation. These inputs inform evidence-based assessments and support proportionate public health decision making.

**Results:**

This collaborative, multi-layered approach facilitates timely and robust assessments that guide targeted mitigation measures, including enhanced surveillance, laboratory testing, clinical readiness, public health guidance, and operational response planning. Recent experiences with pathogens such as Ebola virus, Marburg virus, Lassa fever virus, and mpox demonstrate how integrated intelligence -combining early warning, rapid analysis, and preparedness planning - has informed effective interventions and mitigated potential impacts.

**Conclusions:**

Integrated public health intelligence is a critical pillar of health protection in an interconnected world. By combining early detection, rapid verification, and coordinated action, it enables partners to act decisively before threats escalate and reduces the impact of high-consequence infectious threats nationally and globally. This cohesive system ensures that preparedness and response efforts are timely, evidence-based, and appropriately scaled to risk.

